# 

**Published:** 1891-07-18

**Authors:** 


PRICE ONE PENNY.
No. 251, Vol. X.—July 18th, 1891.
Registered at the General Post Office as a Newspaper for transmission in the United Kingdom and Abroad.]

WITH EXTRA NURSING SUPPLEMENT.

Per Annum, 6s. 6d.; post free.

Monthly Parts, 6d.; post free 8d.

Ditto per Annum, 8s., post free.

### THE HOSPITAL

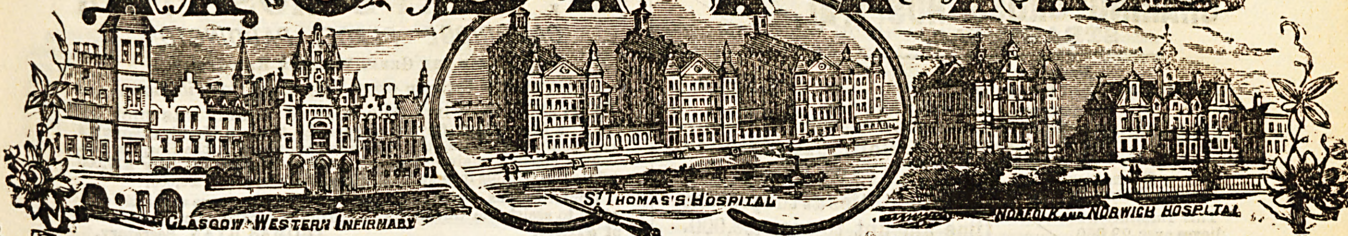

A WEEKLY JOURNAL OF

### Science, Medicine, Nursing and Philanthropy.

### SATURDAY, JULY 18th, 1891.

**Can a Sixpenny Doctor be Honest?** . . . . . 181
**Passing Topics.**—The Duke and the Hospital . . . 182
**The Doctor's Armchair.**—Harvey: An Experimenter . 183
**The History of Capabilities of Herbal Simples.**—By W. T. Fernie, M.D.—XXXV.—Eyebright . . . 184
**Everybody's Page** . . . . . . . 185
**Notes and News** . . . . . . . 183
**General Charities—Scraps and Gleanings** . . . 187
**Annotations.**—"Birch Rods by Post, a Shilling"—Do Jews Ever Plough?—British Fruit and British Farmers . . 188
**The Practitioner's Mirror and Retrospect—**
Medicine.—The Estimation of Hydrochloric Acid in Gastric Disorders, 189; Iodide of Potash . . . . 190
Ophthalmology.—The Rational Treatment of Serous Iritis . . . . . . . . . 190
**The Programme of the Hospital Saturday Fund.**—By Mr. Reginald B. D. Acland . . . . . . 191
**Employments for Women.**—I. Lady Dressmakers . . 192
**Editor's Letter Box.**—Doctors' Fees . . . . 192
**The Student of Medicine.**

### EXTRA SUPPLEMENT—THE NURSING MIRROR.

**En Passant.**—Examinations at the London—Our Friends Abroad—A New Zealand Pro.—Royal National Pension Fund for Nurses—Quiet Nooks—Midwives Again—Short Items—Doctor and Nurse . . . . . . lxxxix

**Lectures on Surgical Ward Work and Nursing.** By Alex. Miles, M.B. Edin., C.M., F.R.C.S.E.—Lecture XXX.—Bandages for the Groin and Perinæum . . . xc

**Nursing Medals and Certificates.**—St. John the Evangelist . . . . . . . . . xci

**The Second Thousand** . . . . . . . xci

**For Reading to the Sick**—Courage . . . . . xci

**Asylum Articles.** III.—The Training of Attendants . xcii

**Cape Colony as a Field of Labour for English Nurses** . . . . . . . . . xcii

**The Princess of Wales' Fund for Mrs. Grimwood** xcii

**Everybody's Opinion.**—District Nursing . . . . xciii

**Wants and Workers—Christmas Competitions** . xciii

**Notes and Queries—Amusements and Relaxation** xciii

**Presentation** . . . . . . . . . xciii

**Off Duty.** In a Devonshire Village . . . . . xciv

